# Polyethylene Storage Tanks Strengthened Externally with Fiber-Reinforced Polymer Laminates

**DOI:** 10.3390/polym17131858

**Published:** 2025-07-03

**Authors:** Ghassan Hachem, Wassim Raphael, Rafic Faddoul

**Affiliations:** Faculty of Engineering and Architecture-ESIB, Saint-Joseph University of Beirut, Beirut 1104 2020, Lebanon

**Keywords:** polyethylene tanks, CFRP strengthening, seismic vulnerability, elephant foot buckling, bonding, surface roughness

## Abstract

Polyethylene storage tanks are widely used for storing water and chemicals due to their lightweight and corrosion-resistant properties. Despite these advantages, their structural performance under seismic conditions remains a concern, mainly because of their low mechanical strength and weak bonding characteristics. In this study, a method of external strengthening using fiber-reinforced polymer (FRP) laminates is proposed and explored. The research involves a combination of laboratory testing on carbon fiber-reinforced polymer (CFRP)-strengthened polyethylene strips and finite element simulations aimed at assessing bond strength, anchorage length, and structural behavior. Results from tensile tests indicate that slippage tends to occur unless the anchorage length exceeds approximately 450 mm. To evaluate surface preparation, grayscale image analysis was used, showing that mechanical sanding increased intensity variation by over 127%, pointing to better bonding potential. Simulation results show that unreinforced tanks under seismic loads display stress levels beyond their elastic limit, along with signs of elephant foot buckling—common in thin-walled cylindrical structures. Applying CFRPs in a full-wrap setup notably reduced these effects. This approach offers a viable alternative to full tank replacement, especially in regions where cost, access, or operational constraints make replacement impractical. The applicability is particularly valuable in seismically active and densely populated areas, where rapid, non-invasive retrofitting is essential. Based on the experimental findings, a simple formula is proposed to estimate the anchorage length required for effective crack repair. Overall, the study demonstrates that CFRP retrofitting, paired with proper surface treatment, can significantly enhance the seismic performance of polyethylene tanks while avoiding costly and disruptive replacement strategies.

## 1. Introduction

Polyethylene (PE) tanks are widely used across industrial and residential sectors for the storage of water, chemicals, and other fluids due to their lightweight nature, corrosion resistance, and cost-effectiveness. Most of these tanks are manufactured using rotational molding techniques and are made of linear or high-density polyethylene grades. While these properties make them attractive for fluid containment, their thin-walled geometry and relatively low mechanical strength raise concerns regarding long-term durability and structural safety, particularly under accidental or seismic loading conditions not considered originally [1,2].

**Figure 1:**
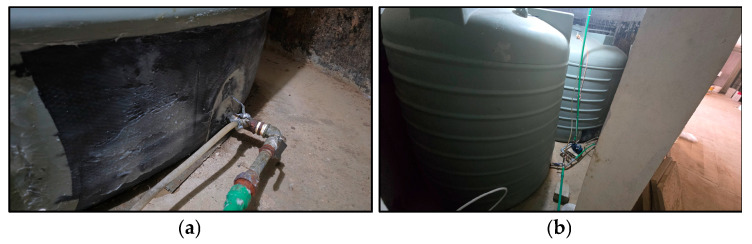
Poly tank strengthening. (**a**) Externally strengthened with CFRPs around outlet nozzle, (**b**) no access for tank replacement.

**Figure 2:**
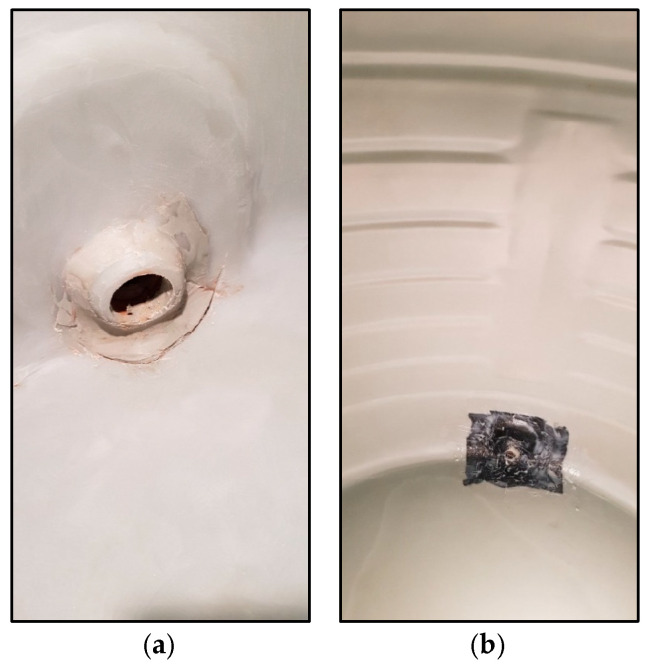
Poly tank cracks around outlet. (**a**) Cracks around outlet nozzle, (**b**) cracks sealed using CFRP laminates.

The limited accessibility of polyethylene tanks installed in confined locations such as basements, rooftops, or integrated piping networks necessitates the adoption of in situ repair and structural strengthening techniques as a practical alternative to complete replacement (Figure 1a,b). When degradation or cracking occurs, caused by ultraviolet (UV) exposure, mechanical impact, or seismic stress, replacement is often costly and logistically difficult, typically requiring full system shutdowns and extensive structural retrofits (Figure 2a,b). Manufacturers typically estimate a service life of 10–15 years [3], but field observations suggest that failure may occur earlier with exposure to dynamic loads or improper support systems.

This paper aims to investigate whether structural retrofitting using fiber-reinforced polymer (FRP) composites can serve as a practical alternative to full tank replacement. FRP systems typically incorporate fibers such as carbon, glass, aramid, or Kevlar, each offering distinct engineering properties—including tensile strength and stiffness—that can be tailored to meet specific application requirements. The performance of the retrofitting system can also be adjusted by varying the number of layers and the total laminate thickness, which commonly ranges from 0.3 mm to 2 mm, depending on the structural demands. FRPs have been successfully implemented in the strengthening of concrete, steel, and masonry structures [4,5]. Studies on metallic tanks have shown that FRP systems can avoid buckling and enhance ductility [5], but these materials have rarely been adapted for polyethylene applications [4]. However, their application to polymer-based structures, particularly polyethylene, remains underexplored due to two key challenges: the inherently low surface energy of polyethylene (~30 mJ/m^2^), which limits adhesive bonding [6,7], and the lack of design-oriented studies that evaluate FRPs’ behavior on thermoplastic substrates under realistic load conditions.

Most technical documents addressing FRP applications focus on steel or concrete substrates [8,9], often overlooking polyethylene due to these bonding limitations. Furthermore, the seismic performance of polyethylene tanks remains insufficiently investigated, particularly concerning critical failure modes such as elephant foot buckling and localized radial deformation. Given the prevalence of polyethylene tanks in densely populated and seismically active regions, addressing this vulnerability through scalable retrofitting methods is of high practical importance. These gaps highlight the need for experimental and numerical studies that evaluate the viability of FRP systems for enhancing the dynamic stability of polymer-based tanks under seismic conditions.

The concept of using CFRPs for structural retrofitting is well established in traditional civil engineering materials, and its application to polyethylene tanks introduces unique scientific challenges. Unlike porous substrates like concrete, polyethylene’s inert surface chemistry, viscoelastic response, and bonding limitations require novel treatment, testing, and modeling strategies. To date, no published studies have systematically assessed the feasibility of CFRP retrofitting on polyethylene tanks using a combined experimental and computational framework.

This approach offers a significant advantage over full replacement, providing a cost-efficient, non-invasive, and rapid strengthening solution suitable for critical infrastructure in space-limited or high-risk environments. This study addresses that gap by proposing and validating a novel FRP retrofitting methodology for polyethylene tanks. A hybrid experimental–numerical approach is used to assess bond behavior, anchorage requirements, and structural performance under seismic loading. First, tensile tests are conducted on CFRP-reinforced polyethylene strips to quantify bond strength and failure modes. Surface roughness analysis is performed using image-based pixel intensity techniques to characterize the effects of mechanical grinding. Second, finite element analysis (FEA) is conducted using ABAQUS [10] to simulate full-scale tank behavior under hydrostatic and seismic loading, with and without CFRP wrapping. The results are used to propose an anchorage length formula tailored for polyethylene applications and to evaluate the validity of modeling CFRP–polyethylene interaction through a tied connection.

By integrating experimental validation, image-based surface analysis, and nonlinear seismic modeling, this research presents one of the first comprehensive evaluations of CFRP retrofitting for thermoplastic storage tanks. The outcomes not only contribute to the scientific understanding of polymer–FRP systems, but also provide urgently needed retrofit strategies for aging polyethylene infrastructure in urban, hazard-prone areas.

## 2. Literature Review

Carbon fiber-reinforced polymer (CFRP) composites are extensively used in retrofitting civil infrastructure because of their excellent tensile strength, low weight, and corrosion resistance, and they are increasingly cost-competitive with metals [11,12]. Their effectiveness in improving the seismic performance of reinforced concrete structures has been well demonstrated, particularly in circular tanks where both the flexural and shear capacity are significantly enhanced [13]. Likewise, CFRPs have been implemented in large-scale applications such as wind turbine towers and industrial chimneys, where dynamic and environmental loads necessitate lightweight and durable retrofitting systems [14].

Cylindrical shells such as tanks, silos, and pipes can benefit substantially from CFRP reinforcement, especially when subjected to internal pressure or dynamic lateral forces. Experimental and numerical investigations have shown that CFRP wrapping mitigates local buckling, improves load distribution, and increases the energy dissipation capacity [4]. These benefits are particularly important under seismic loading, where thin-walled cylindrical shells are prone to failure modes such as elephant foot buckling and radial deformation [13].

Polyethylene (PE) tanks are widely used due to their corrosion resistance, chemical compatibility, and ease of manufacturing through rotational molding [1]. However, their mechanical limitations—such as poor tensile strength and high deformability—make them vulnerable under seismic loads [2]. Thin-walled PE shells are susceptible to hoop stress, base uplift, and local instability, especially when subjected to changes in fluid density or earthquake-induced accelerations. Although standards like API 650 Appendix E [15] offer seismic design guidance for steel tanks, no established seismic retrofit methodology exists for polyethylene tanks. Peer-reviewed studies on the CFRP retrofit of PE tanks remain limited, despite the presence of numerous technical documents and industry reports that recommend reinforcement and repairs involving fiberglass cloth, acrylic canvas, and particularly stainless steel mesh embedded in welded or polyurethane-based repairs [16,17]. These methods conceptually resemble fiber-reinforced polymer (FRP) systems, where reinforcing fibers are embedded in adhesives.

Bonding CFRP to polyethylene presents a critical challenge due to the inherently low surface energy of PE (~30 mJ/m^2^) [6,7]. This prevents good wetting by adhesives and limits chemical interaction. Conventional repair methods involve thermal welding, where the polymer chains in the base material and filler re-melt and fuse into a continuous mass. While this method ensures chemical compatibility, it often introduces residual stresses due to rapid heating and cooling and produces joints that are weaker than the base material in tensile tests [16,17]. Additionally, field-welded joints tend to be inconsistent, especially when operator experience or environmental conditions vary [16,17].

Patches or fillers applied using epoxy or MMA adhesives have been used to restore tanks considered irreparable [18]. However, bonding to polyethylene is inherently difficult due to its non-polar and chemically inert nature [19]. Without surface treatment, adhesives yield very low shear strength on polyolefins. For example, a general-purpose epoxy achieved a shear strength of less than 1 MPa on untreated polyethylene, but this value approximately doubled with plasma pretreatment [20,21]. Surface activation techniques such as flame, corona, or plasma treatment are often used to oxidize the surface and improve adhesion [20]. Nonetheless, adhesive bonds generally remain weaker than welds and raise durability concerns in wet or chemical environments.

Other repair techniques—such as embedding stainless steel wire mesh across a crack prior to welding over it or installing mechanical flanges or bolted patches—have been employed in practical applications to restore structural integrity [20,21]. However, these are rarely documented in the scientific literature. Mechanical fastening creates stress concentrations, requires drilling (introducing new weak points), and does not guarantee watertightness. Moreover, mechanical solutions add weight and may compromise the tank’s flexibility, which is one of polyethylene’s key design advantages.

Creep is another critical concern in polyethylene (PE) storage tanks, as the material exhibits time-dependent deformation under sustained loads. Neglecting creep in structural applications can compromise long-term performance and safety. In civil engineering—particularly in concrete structures—creep is a well-recognized phenomenon, necessitating accurate modeling to ensure durability and serviceability [22]. In PE tanks, creep may lead to gradual stretching, bulging, or the buckling of shell walls. Without appropriate design considerations, this may lead to cracking or leakage, posing significant risks in high-stakes storage applications such as acids or wastewater [23]. While various solutions have been proposed to mitigate creep in concrete structures, including design-based approaches and material enhancements [22], comparable strategies have not yet been developed for polyethylene members [24,25]. Despite the well-documented role of creep in the long-term deformation and potential failure of PE tanks, the literature remains largely silent on effective reinforcement methods tailored to this material [26]. Localized deformations—such as nozzle sagging—may be corrected through minor interventions, but global creep-induced distortions require more robust reinforcement strategies.

Although no technical documents or industrial case studies have directly addressed this issue for PE tanks [24,25], the application of external CFRP wrapping to mitigate creep has shown promising results in other infrastructure systems. CFRP wraps act as high-modulus external layers that increase shell stiffness, resist hoop stresses, redistribute loads, and seal pre-existing cracks. These benefits align with successful applications in the strengthening of concrete and metallic tanks [4,5].

Over the last decade, the decreasing cost of CFRPs and epoxy systems has expanded their application beyond niche retrofits. CFRPs are now considered economically viable for large-scale projects, especially when lifecycle performance and downtime savings are included in the cost assessment [11,12]. Del Vecchio et al. [11] showed that CFRP retrofit solutions offer an excellent cost-to-performance ratio in the seismic rehabilitation of reinforced concrete buildings. Other studies have also highlighted the economic competitiveness of carbon composites compared to metals in structural applications, especially when factoring in weight, corrosion resistance, and ease of installation [12].

Recent advancements in surface roughness evaluation have highlighted the efficacy of image-based techniques in evaluating the surface preparation of polyethylene for proper bonding to CFRPs [27,28,29]. Combining pixel intensity and texture features, such as standard deviation filtering, enhances image segmentation accuracy, which is pivotal for surface characterization [27]. Abas et al. [28] analyzed surface roughness variations in nylon carbon fiber composites fabricated via fused deposition modeling, emphasizing the influence of the process parameters on the surface quality. Simunovic et al. [29] used digital image analysis to assess machined surface roughness, establishing a correlation between image-derived metrics and traditional roughness parameters. Collectively, these studies underscore the potential of integrating image processing methods for accurate and efficient surface roughness assessment.

To address these challenges, this study integrates experimental anchorage testing, surface roughness analysis, and full-scale finite element modeling. The goal is to assess the feasibility of CFRP retrofitting for PE tanks and to propose design-oriented solutions, including anchorage length formulations.

## 3. Experimental Methodology

**Figure 3:**
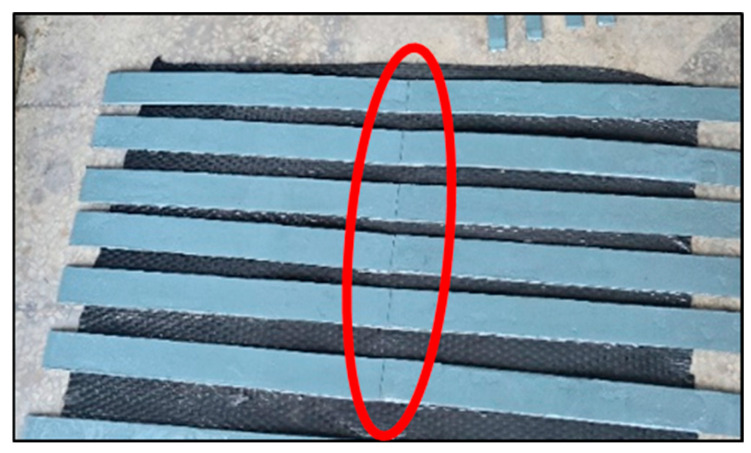
Butt placement of the polyethylene strips before CFRP application on both sides.

To investigate the bond behavior between CFRPs and polyethylene, tensile tests were conducted on polyethylene strips reinforced with CFRP laminates on both sides. The specimens were prepared in a butt joint configuration without any mechanical fasteners, simulating a typical crack repair scenario in tank walls (Figure 3). The goal was to determine the anchorage length required to prevent slippage and to evaluate the failure modes, including cohesive failure in the substrate and interfacial slip. Failure of the CFRP itself was not considered, given the significantly higher strength of the composite material compared to polyethylene. It is important to note that the test setup effectively simulates a through crack in the tank wall. This configuration mimicked a typical structural discontinuity or damage scenario.

**Figure 4:**
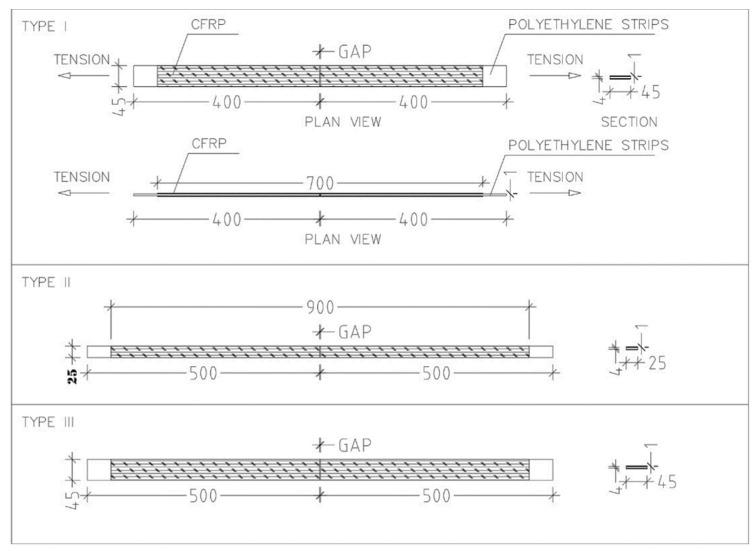
Specimen details.

Three specimen types were extracted from an actual cylindrical polyethylene tank manufactured via rotational molding. All strips were 4 mm-thick linear low-density polyethylene (LLDPE). Type I measured 800 mm in length and 45 mm in width, with a 700 mm CFRP laminate length; Type II had a length of 1000 mm, a width of 25 mm, and a 900 mm CFRP laminate; and Type III was 1000 mm long, 45 mm wide, and included a 900 mm CFRP laminate. Each configuration was tested five times to assess consistency and variability across geometries (Figure 4).

**Figure 5:**
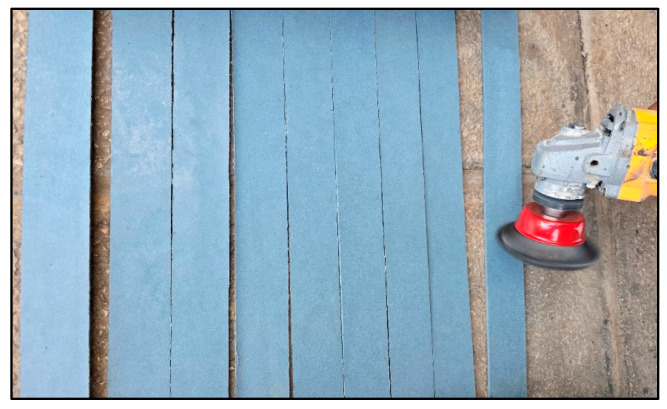
Surface scarification of polyethylene strips using mechanical grinder.

To enhance bonding performance, all polyethylene surfaces were mechanically scarified using a rotary grinder, following ISO 17212 guidelines for plastic surface preparation [30]. This treatment aimed to increase the surface energy and micro-texture, both critical factors for adhesive bonding on polyolefins (Figure 5).

**Figure 6:**
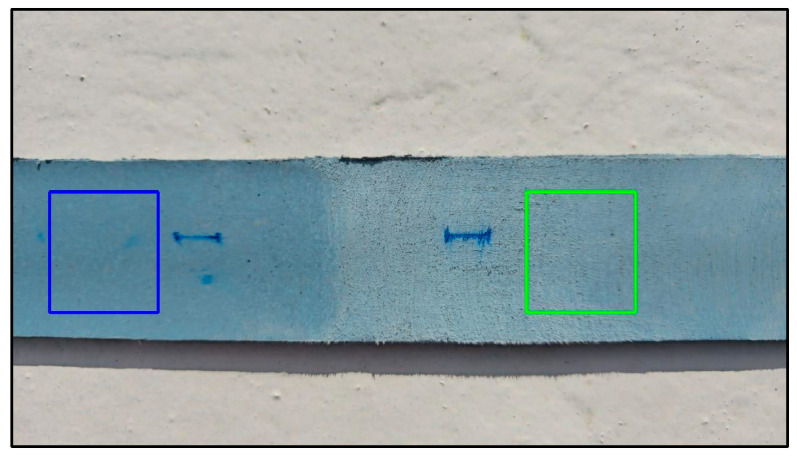
Polyethylene strip with analysis zones: blue marks untreated, green marks treated; areas inside squares were analyzed for surface roughness.

To assess the effectiveness of surface preparation, a field-adaptable grayscale image analysis method was employed. While this technique does not yield absolute roughness metrics, it serves to complement visual inspection in situations where standard instruments—such as profilometers—are unavailable. Smartphone images were captured of both treated and untreated regions, with a 1 cm marker included for spatial calibration (Figure 6).

**Figure 7:**
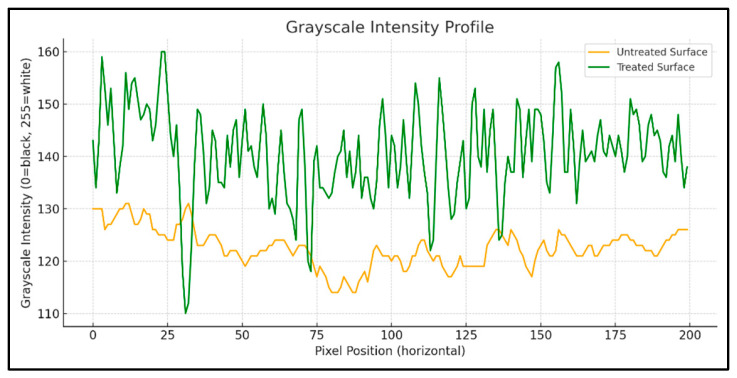
Grayscale intensity profile.

**Figure 8:**
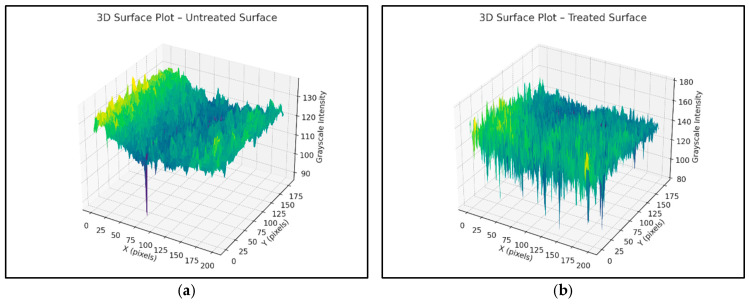
(**a**) Untreated polyethylene 3D surface. (**b**) Treated polyethylene 3D surface.

Grayscale intensity values across selected zones were extracted using Python 3.10 libraries (NumPy, OpenCV, PIL, and Matplotlib). The treated zone exhibits an increase in grayscale intensity standard deviation, confirming a measurable enhancement in surface roughness. These results are visualized using 3D grayscale surface plots, clearly illustrating an enhanced texture in the sanded regions (Figure 7 and Figure 8a,b).

**Figure 9:**
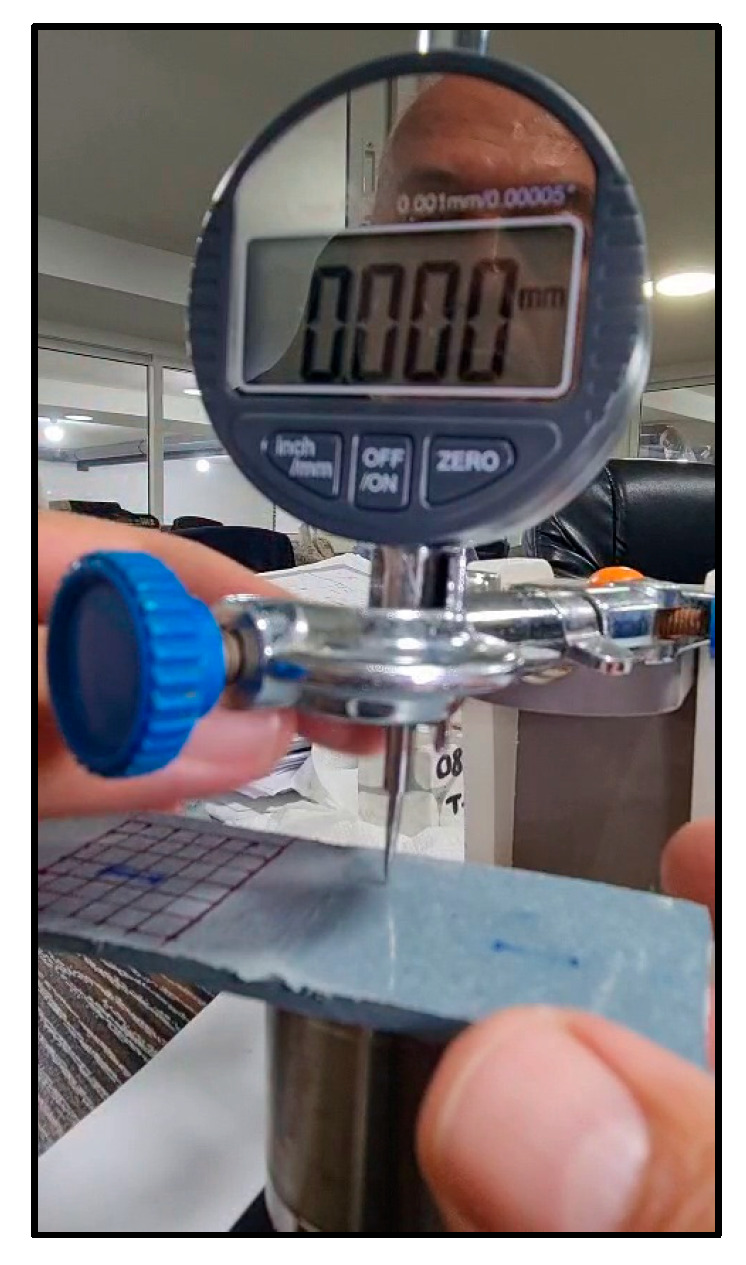
Contact profilometer measuring surface roughness (Ra) on polyethylene specimen.

To validate the grayscale-based surface assessment, profilometry measurements were conducted on the sanded region using a contact-type surface profilometer (Figure 9). The average roughness (Ra) was measured at approximately 20 µm, confirming a substantial increase in the surface texture due to mechanical scarification. This observation aligns with grayscale intensity variations detected through image analysis, and corresponds to the roughness levels reported in prior studies. Abas et al. (2023) referenced Ra values of ~21 µm for polymer composites and highlighted a positive correlation between grayscale intensity and surface roughness in material extrusion manufacturing processes [28]. The integration of grayscale imaging and profilometry enhances the reliability of surface characterization for adhesive bonding applications.

**Figure 10:**
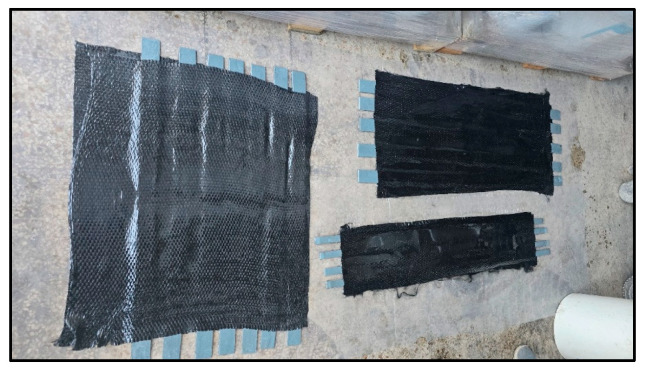
CFRP laminate placement—wet application.

**Figure 11:**
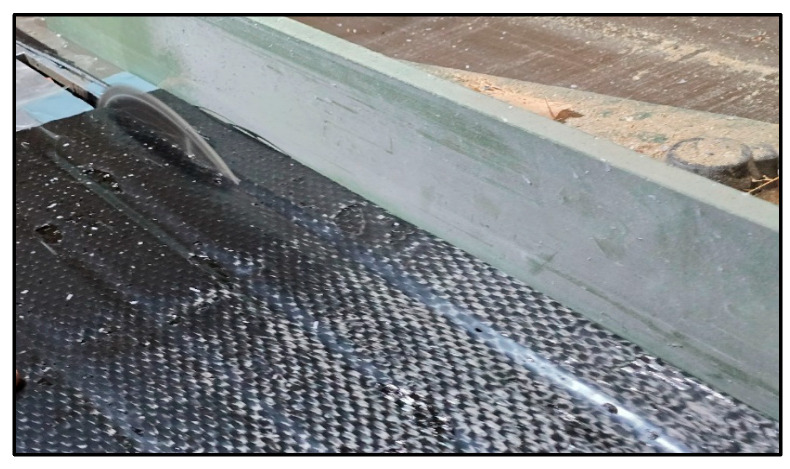
Saw cut of the CFRP/poly specimens.

**Figure 12:**
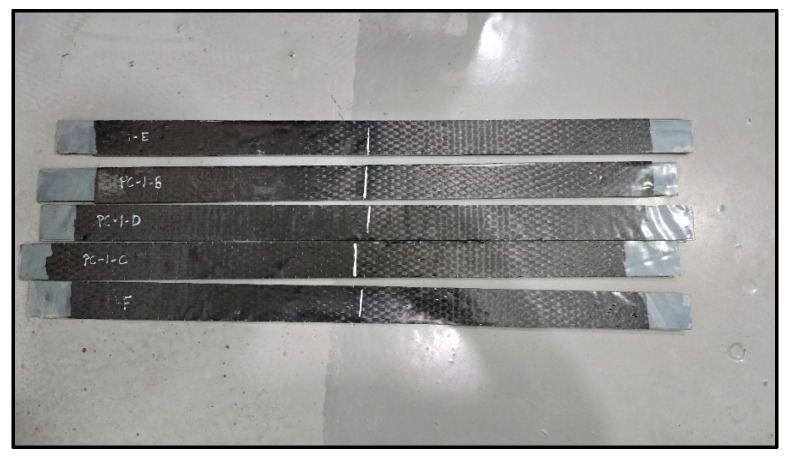
Specimens.

CFRP laminates were wet-applied to the scarified polyethylene strips using TYFO S epoxy resin from FYFE Co. [31], a system formulated for structural FRP bonding. The fibers were oriented longitudinally along the axis of each specimen. To ensure symmetry and avoid eccentric mesh during testing, one layer of CFRP was applied on each side. After curing, the specimens were trimmed to size using a mechanical saw, and CFRP coverage was verified to fully cover the butt joint region (Figure 10, Figure 11 and Figure 12).

**Figure 13:**
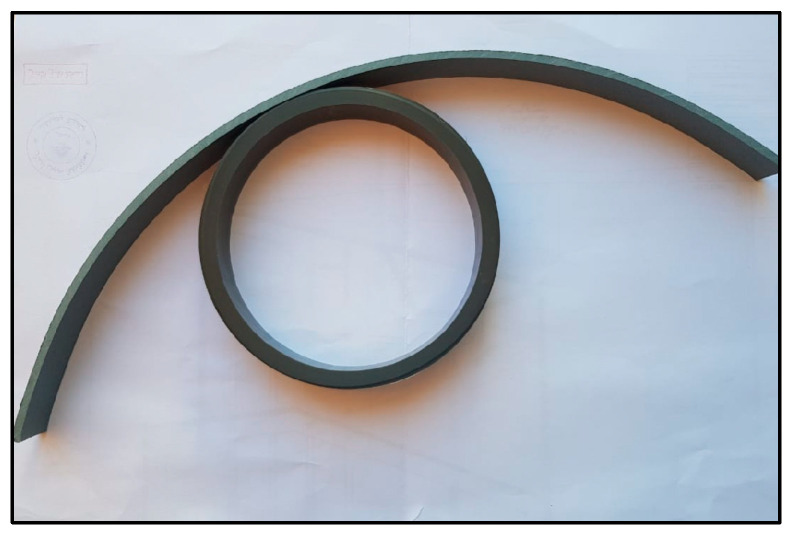
Placement of polyethylene strip around the cylindrical jig.

**Figure 14:**
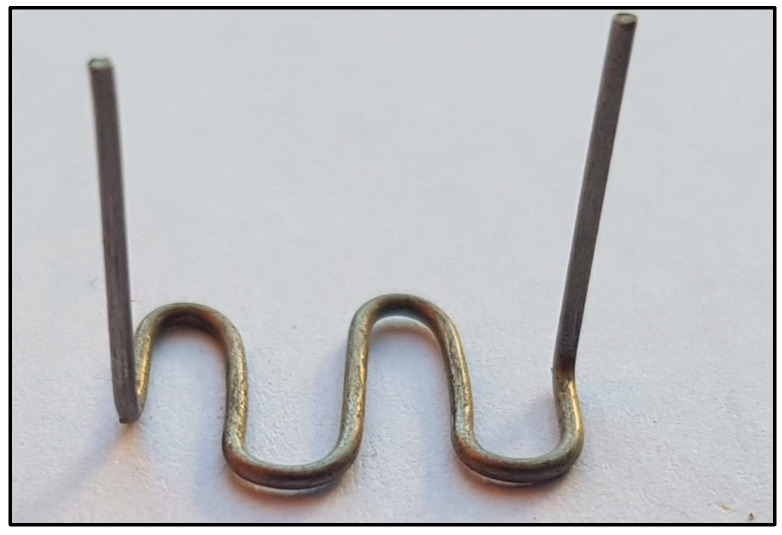
Steel connector used to secure the polyethylene ring during CFRP application.

**Figure 15:**
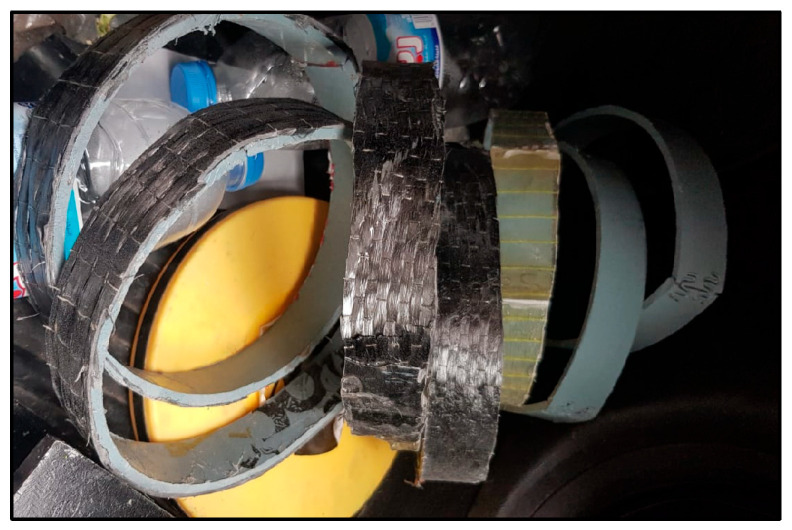
Completed CFRP wrapping on curved polyethylene specimen.

**Figure 16:**
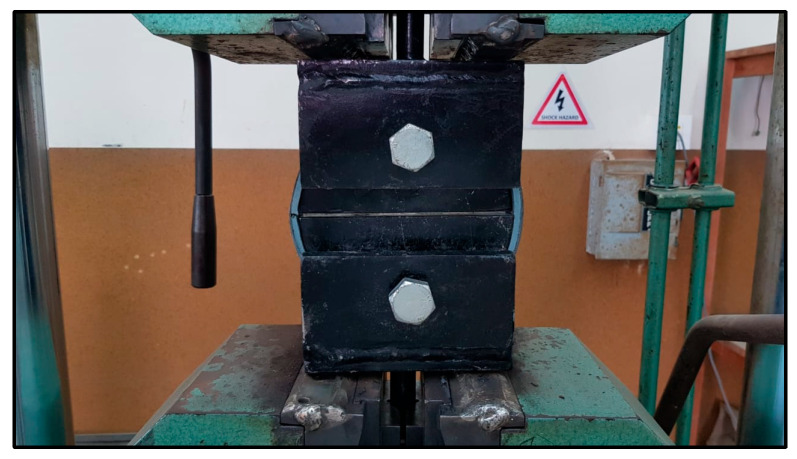
Split-disk test.

Prior to the flat strip tensile tests, a series of five tensile ring tests were conducted following the ASTM D2290 split-disk method to simulate circumferential loading conditions in polyethylene tanks [32]. To create ring specimens, polyethylene strips were formed around a thick plastic jig fabricated from a segment of a plastic pipe to match the split-disk curvature (Figure 13). The strip ends were secured using zigzag steel connectors to retain the circular geometry during CFRP application and curing (Figure 14). After curing, the CFRP-wrapped rings were subjected to tensile loading using a split-disk fixture mounted in a universal testing machine (Figure 15 and Figure 16).

**Figure 17:**
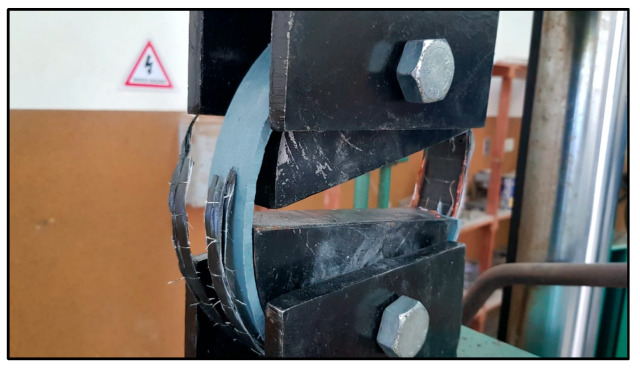
Tensile failure of CFRP layer during split-disk test.

The CFRP laminates were applied externally in a continuous circumferential wrap to simulate field application. However, all ring specimens failed due to the tensile rupture of the CFRP prior to any significant deformation or slippage at the CFRP–polyethylene interface (Figure 17). This is attributed to the mismatch in elongation capacity: CFRP fails at a ~1.5% strain, while LLDPE can elongate beyond 300–500%.

Thus, the ring tests were effective in confirming CFRPs’ tensile behavior, but provided limited insight into interfacial bond performance or anchorage behavior. The flat strip tests were therefore retained as the primary method for bond evaluation.

The polyethylene used in this study is a SABIC [33] LLDPE grade formulated for rotational molding, with an elastic modulus of 1.05 GPa, a yield tensile strength of 16 MPa, and CFRP properties provided by FYFE Co. [31], including a tensile modulus of 95.8 GPa and ultimate tensile strength of 986 MPa.

The full orthotropic properties are summarized in Table 1. The laminate thickness is 1 mm, and all tensile tests are performed under displacement control using a universal testing machine.

These experimental results are used to validate a proposed anchorage length design model and to justify the use of tied contact in the finite element modeling phase.

## 4. Results and Discussion of Experimental Tests

The grayscale image analysis shows that mechanical sanding substantially modifies the surface topography of polyethylene. The standard deviation of grayscale intensity increased by approximately 127% in treated zones; suggesting significantly higher surface roughness, conducive to improved adhesive bonding.

**Figure 18:**
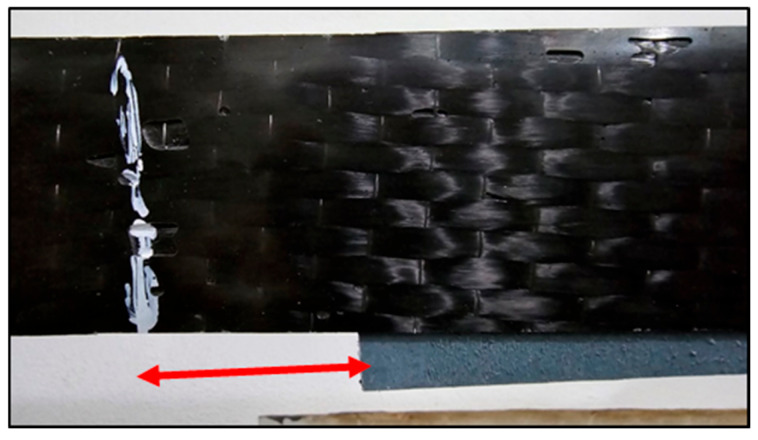
Slippage (side view) of the CFRP.

**Figure 19:**
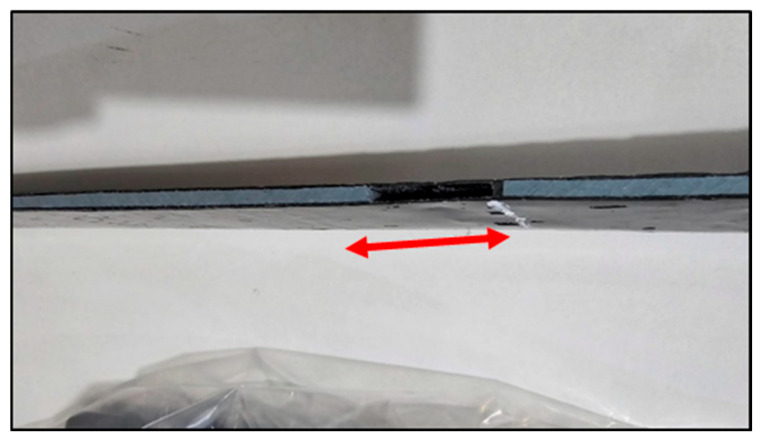
Slippage (top view) of the CFRP.

**Figure 20:**
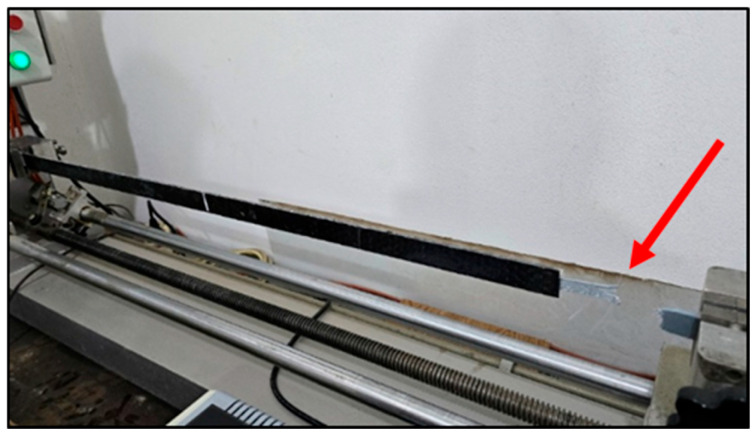
Tensile Test—yielding of polyethylene strips.

Tensile bond tests revealed that the shear bond strengths between CFRP and polyethylene ranged from 0.064 to 0.091 MPa, depending on laminate width and length. These results suggest that CFRP laminates can be used to seal cracks in polyethylene tank shells, provided that an adequate anchorage length and surface preparation are ensured. Out of 15 total specimens, 8 showed interfacial slippage, primarily in Type I and II configurations (Figure 18 and Figure 19), and 7 exhibited failure in the plastic, primarily in Type III configurations (Figure 20).

The failure modes were categorized as follows: Mode 1—plastic failure in Type III where the polyethylene strips ruptured before CFRP delamination (Figure 20). Mode 2—interface slippage: seen in eight tests from Types I and II, where the CFRP began to de-bond before the full tensile capacity was reached (Figure 18 and Figure 19).

When the anchorage length exceeded approximately 450 mm, failure consistently occurred within the polyethylene substrate rather than at the adhesive interface. This transition from interface slippage to base material failure suggests that the CFRP’s reinforcement effectively bridged and sealed the simulated crack, restoring structural continuity. These results reinforce the potential of CFRP laminates as a viable in situ sealing method for real-life polyethylene tank cracks, provided that an adequate anchorage length is ensured.

**Figure 21:**
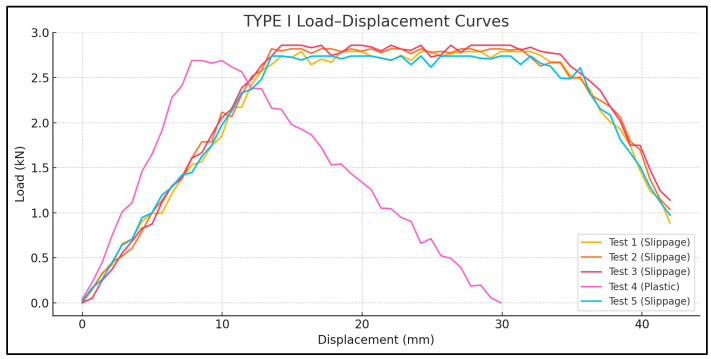
Load displacement diagram—Lplastic 800 mm, LCFRP 700 mm, and Wplastic 45 mm.

**Figure 22:**
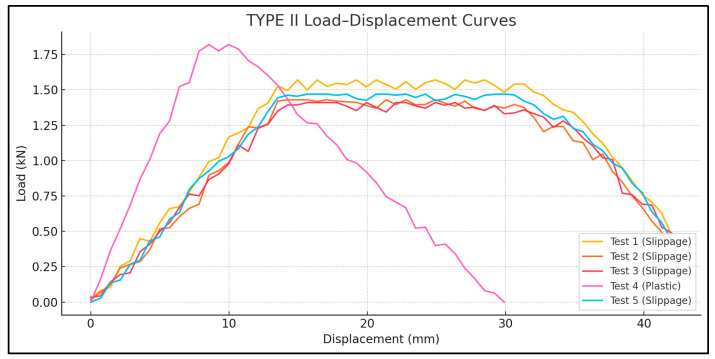
Load displacement diagram—Lplastic 1000 mm, LCFRP 900 mm, and Wplastic 25 mm.

**Figure 23:**
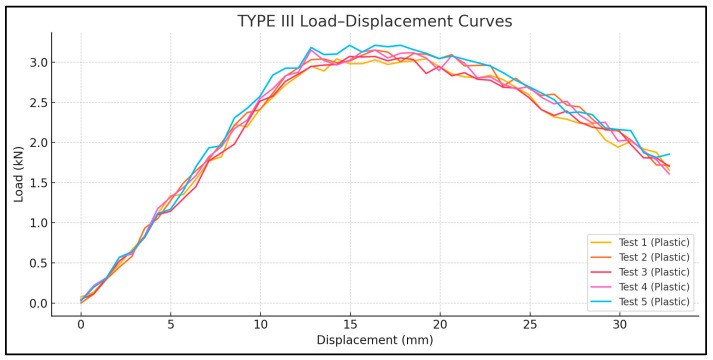
Load displacement diagram—Lplastic 1000 mm, LCFRP 900 mm, and Wplastic 45 mm.

The load–displacement curves (Figure 21, Figure 22 and Figure 23) reveal distinct differences in failure behavior across the specimen types. Type I and Type II specimens exhibited pronounced plateaus in the load response—characteristic of slippage failure—whereas Type III showed a linear load increase followed by a sharp drop, indicative of plastic rupture.

The maximum tensile forces and shear bond values are summarized in Table 2 and Table 3.

For Type I, the tensile loads averaged 2.78 KN, with one outlier showing plastic failure at 2.69 KN and a coefficient of variation (CoV 2.4%) indicating that a sufficient laminate width can play a key role in achieving uniform bond quality. For Type II, the average tensile capacity is 1.54 KN, lower due to the reduced bond area, though one test reached 1.82 KN with substrate rupture. The high variability in Type II (CoV 10.9%) can be attributed to its narrower CFRP width (25 mm), which may increase sensitivity to bonding inconsistencies or adhesive coverage errors. Type III exhibited the highest tensile loads, ranging from 3.04 to 3.21 KN, suggesting that an increased bond area enables more effective stress transfer, achieving a complete bond and preventing any slippage; hence, reaching plastic failure and a CoV of 2.2%.

A comparison of Type I and III—both with a 45 mm CFRP width—demonstrates that a longer laminate length (700 mm vs. 900 mm) significantly enhances bond reliability. This is evident in the failure modes: Type III consistently exhibited plastic rupture, while Type I showed interface failure in four out of five cases.

To generalize the test findings for design purposes, an anchorage length model is developed based on the equilibrium between tensile and bond shear forces. The total tensile capacity of the plastic substrate is expressed as the following:

T_s_ = T_b_ (1)

where T_s_ is the tensile force in the plastic substrate at ultimate capacity and T_b_ is the total bond force developed at the interface between the CFRP and plastic. Considering that the substrate tensile force at failure, T_s_, depends on the tensile strength of the plastic σ_p_, the thickness of the plastic t_p_, and the effective width under tension w_p_, T_s_ can be expressed as follows:

(2) $$T_{s}=\sigma_{p}\left(t_{p}\cdot w_{p}\right)$$

Since the CFRP is symmetrically bonded on both sides, each side of the FRP reinforcement contributes equally to resisting the tensile force from the plastic substrate. Thus, the tensile force is distributed evenly, and each interface must transfer half of the total tensile load. Therefore, the force equilibrium formula can be expressed as follows:

(3) $$\sigma_{p}\left(t_{p}\cdot w_{p}\right)=2\left(\tau_{b}\cdot L_{anch}\cdot w_{p}\cdot C_{p}\right)$$

The right side of Equation (3) represents the total resisting bond force on both FRP–plastic interfaces, where τ_b_ is the average bond shear stress at the FRP–plastic interface, L_anch_ is the anchorage length, and C_p_ is an empirical calibration factor introduced based on experimental data to account for practical conditions such as surface preparation quality, adhesive characteristics, and curing conditions.

By canceling out the effective width of plastic, or crack width w_p_, as it appears on both sides, the expression simplifies to the anchorage length formula provided in Equation (4):

(4) $$L_{anch}=\frac{\sigma_{p}\cdot t_{p}}{2\cdot \tau_{b}\cdot C_{p}}$$

An explicit safety factor γ_s_ is further considered to account for bond imperfections due to joint assembly. A recommended value of 2 is proposed, reducing the allowable bond shear stress. This explicit inclusion ensures conservative and safe design, significantly increasing structural reliability.

The recommended anchorage length to safely seal and connect two polyethylene strips is defined in Equation (5):

(5) $$L_{anch}=\frac{\sigma_{p}\cdot t_{p\cdot }\gamma_{s}}{2\cdot \tau_{b}\cdot C_{p}}$$

Given that the full wrapping of tanks is common in practice, with the CFRP extending 200 mm beyond the starting wrapping point, this justifies the use of tied contact in FEA simulations. This assumes a continuous, no-slip interface between the CFRP and polyethylene—an assumption justified by the experimental results.

## 5. Finite Elements Analysis

**Figure 24:**
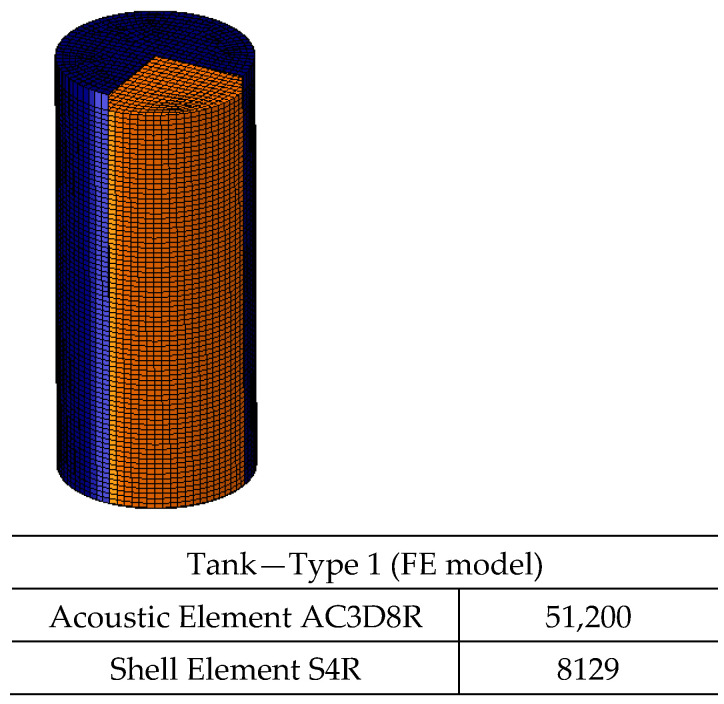
Tank Type 1.

**Figure 25:**
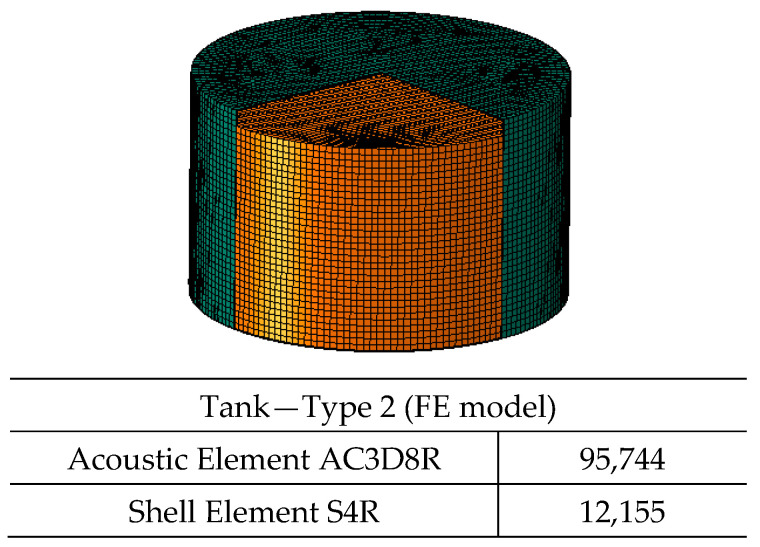
Tank Type 2.

Two types of water tanks are modeled in ABAQUS [10] under hydrostatic and seismic loading conditions (Figure 24 and Figure 25). The tank dimensions and shell thicknesses are based on commercially available tanks [34], with diameter-to-height ratios (D/H) either below or above the 1.333 threshold specified in API 650 Appendix E [15].

The tanks’ shell thicknesses (T) used in the models are calculated according to the design requirements of ASTM D1998 (Standard Specification for Polyethylene Upright Storage Tanks) [35]:

(6) $$T=\frac{P\cdot D}{2\cdot SD}$$

where T is the wall thickness (mm), P is the fluid pressure at the shell bottom (MPa), and SD is the hydrostatic design stress (MPa). The tank dimensions and computed thicknesses are summarized in Table 4.

**Figure 26:**
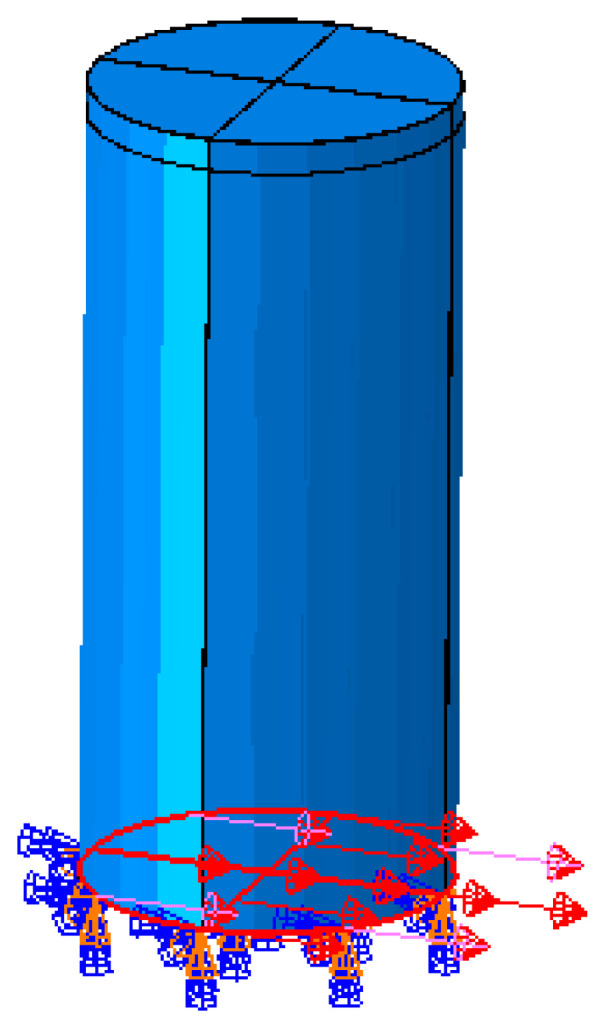
Base fixed only allowed in X direction.

**Figure 27:**
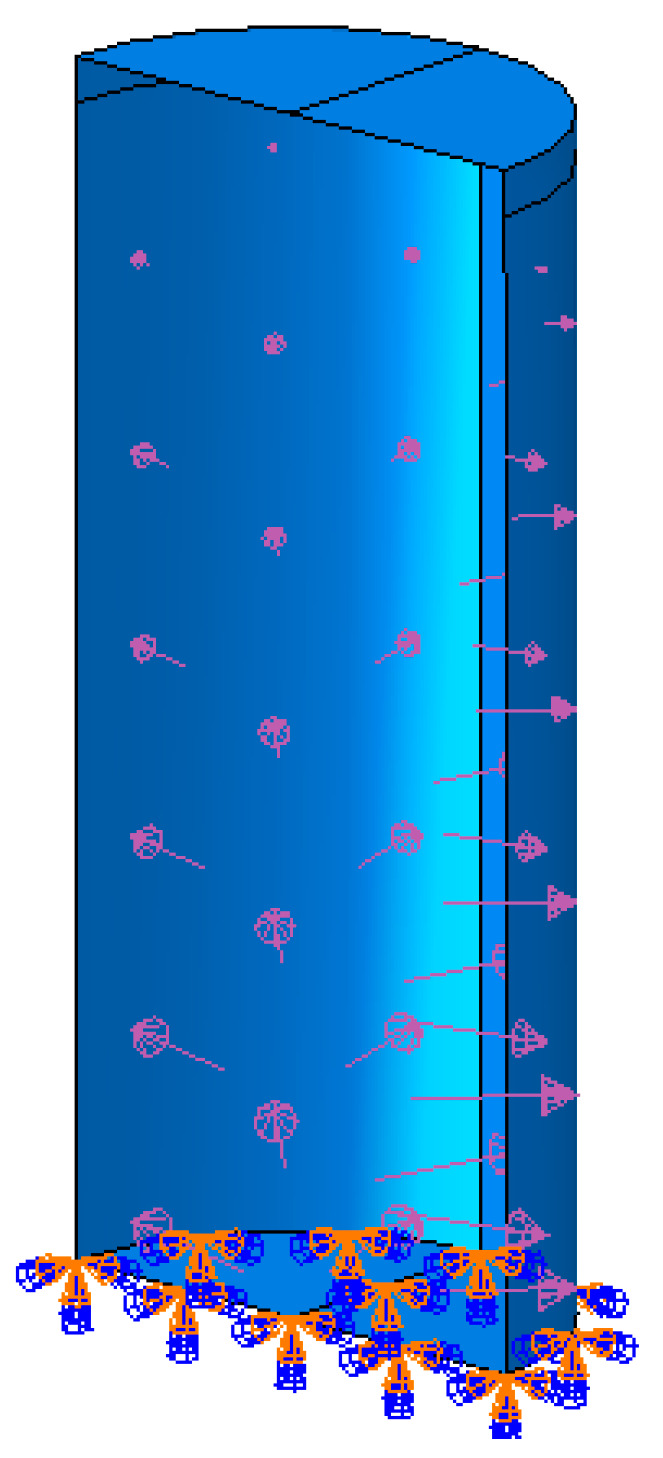
Hydrostatic.

A service factor of 0.5 is applied to account for safety, as recommended by ASTM D1998 [36]. The hydrostatic loading conditions are illustrated in Figure 26 and Figure 27.

Simulations were conducted to evaluate the stress distributions in polyethylene tanks subjected to both hydrostatic and seismic loading, with and without external carbon fiber-reinforced polymer (CFRP) reinforcement. The seismic load applied in this analysis is based on the Kobe earthquake, with a peak ground acceleration of 0.3 g. Externally bonded CFRP laminates are applied to reduce deformation and limit peak stresses, as shown in the Appendix A.

## 6. Cost Analysis

This section assesses the economic and structural merits of externally retrofitting polyethylene tanks with fiber-reinforced polymer (FRP) laminates versus full tank replacement. FRP retrofitting is shown to be both cost-efficient and technically effective, utilizing the existing tank shell while significantly enhancing structural performance and resilience. This approach mitigates degradation phenomena over time and extends service life without major operational disruptions.

From a structural engineering perspective, applying unidirectional carbon fiber externally enhances the stiffness and integrity of the tank shell, particularly under long-term loads and environmental exposure. This retrofit strategy is practical for facilities requiring uninterrupted operation, and is especially relevant in seismically active regions where conventional replacement may not be feasible within limited time frames.

Additionally, it is important to note that the presented analysis is based on the use of unidirectional carbon fiber, which represents a high-performance but comparatively high-cost reinforcement option. If glass fiber fabrics were utilized instead—an alternative with a substantially reduced cost—the overall strengthening expense could be further decreased. Moreover, the unit price of fiber materials tends to decrease significantly when procured in bulk quantities, making FRP strengthening even more cost-competitive for large-scale applications. The cost figures presented in Table 5 are reflective of the market conditions in the Kingdom of Saudi Arabia (KSA) at the time of assessment, and may vary depending on regional pricing structures and supply chain dynamics.

The cost analysis clearly demonstrates the significant economic advantage of fiber-reinforced polymer (FRP) strengthening over complete tank replacement. For Tank Type 1 (D = 2.35 m, H = 5.2 m), the total cost of FRP strengthening is USD 1152, whereas the cost of complete replacement is USD 3200. Similarly, for Tank Type 2 (D = 4.5 m, H = 2.7 m), FRP strengthening costs USD 1146 compared to USD 5050 for a full replacement. These results highlight the substantial cost savings associated with the adoption of FRP strengthening methods, reducing initial investment and minimizing operational downtime. Consequently, FRP strengthening not only ensures improved structural durability and seismic performance, but also offers significant economic and operational benefits compared to traditional tank replacement [36].

## 7. Conclusions

This study investigated the feasibility of strengthening polyethylene tanks using externally bonded CFRP laminates, combining experimental testing with finite element modeling. The sealing performance of CFRPs at crack locations was evaluated, and a design-oriented formula was proposed for determining the required anchorage length in butt joint configurations. The finite element results further demonstrated the effectiveness of CFRP wrapping in reducing hoop stresses under seismic loading conditions. These findings underscore the potential of CFRP retrofitting as a practical, non-invasive alternative to full tank replacement, particularly in seismically active or densely populated regions where rapid intervention and limited physical access are critical considerations.

Future research may explore alternative reinforcement materials, such as glass fiber, to assess cost-effectiveness and compatibility with polyethylene substrates. Additionally, we recommend incorporating standardized roughness parameters (e.g., Ra or Sa) using contact profilometry in future studies, to validate and correlate grayscale-based surface assessments.

## Figures and Tables

**Table 1:** Properties of CFRP sheet reinforcement.

CFRP Material Properties ($t_{f}$ = 1 mm)						
Data	E_1_	E_2_	Nu_12_	G_12_	G_13_	G_23_
Units	Mpa	Mpa		Mpa	Mpa	Mpa
Value	95,800	5101	0.216	1980	1220	1220
Data	Longitudinal Tensile Strength	Longitudinal Compressive Strength	Transverse Tensile Strength	Transverse Compressive Strength	Longitudinal Shear Strength	Transverse Shear Strength
Units	Mpa	Mpa	Mpa	Mpa	Mpa	Mpa
Value	986	455	72	80	30	30

Note: E_1_ and E_2_ are Young’s modulus in the fiber and transverse directions, respectively. Nu_12_ is the major Poisson’s ratio. G_12_, G_13_, and G_23_ represent the in-plane and out-of-plane shear modulus. These parameters define the orthotropic elastic behavior of the composite, as required in Abaqus [10] simulations under plane stress conditions.

**Table 2:** Maximum tensile and stress values for each test.

	Test 1 (KN)	Test 2 (KN)	Test 3 (KN)	Test 4 (KN)	Test 5 (KN)	Average Tensile Force (KN)	Bond Area (mm^2^)	Net Poly Section (mm^2^)	Stress in Poly Strip Test 1	Stress in Poly Strip Test 2	Stress in Poly Strip Test 3	Stress in Poly Strip Test 4	Stress in Poly Strip Test 5
TYPE I: Lpoly 800 mm, LCFRP 700 mm, Wpoly 45 mm	2.78	2.82	2.86	2.69	2.74	2.78	3150	180	15.44	15.67	15.89	14.94	15.22
TYPE II: Lpoly 1000 mm, LCFRP 900 mm, Wpoly 25 mm	1.57	1.43	1.41	1.82	1.47	1.54	2250	100	15.70	14.30	14.10	18.20	14.70
TYPE III: Lpoly 1000 mm, LCFRP 900 mm, Wpoly 45 mm	3.04	3.15	3.07	3.15	3.21	3.12	4050	180	16.89	17.50	17.06	17.50	17.83

**Table 3:** Shear bond values.

	Shear Bond Values for Each Test Before Slippage or Poly Failure (MPa)					
	Test 1	Test 2	Test 3	Test 4	Test 5	Average Bond (MPa)
TYPE I: Lpoly 800 mm, LCFRP 700 mm, Wpoly 45 mm	0.088	0.090	0.091	0.085 *	0.087	0.088
TYPE II: Lpoly 1000 mm, LCFRP 900 mm, Wpoly 25 mm	0.070	0.064	0.063	0.081 *	0.065	0.068
TYPE III: Lpoly 1000 mm, LCFRP 900 mm, Wpoly 45 mm	0.075 *	0.078 *	0.076 *	0.078 *	0.079	0.077

* Polyethylene failure.

**Table 4:** Dimensions and calculated shell thickness of polyethylene tanks.

Tank	D (Tank Diameter) (mm)	H (Fluid Head) (m)	D/H	SD (Mpa)	Pressure (Mpa)	Calculated Thickness (mm)	Tank Shell Thickness (mm)
Type 1—Tall Tank	2350	5200	0.45	8	0.051	7.49	8
Type 2—Shallow Tank	4500	2700	1.67	8	0.026	7.45	8

**Table 5:** Comparative table—Tank Replacement versus Tank Shell Strengthening.

Description of Activities	Tank Type 1 (D = 2.35 m, H = 5.2 m)	Tank Type 2 (D = 4.5 m, H = 2.7 m)
Option—Tank Replacement		
Cost of New Tank (USD)	2500	4250
Cost of Mechanical Connections (removal/reinstallation) (USD)	300	300
Cost due to Operational Downtime (USD)	Depending on plant activities	Depending on plant activities
Cost of Transportation and Crane Usage (USD)	400	500
Total Tank Replacement Cost (USD)	3200	5050
Option—Tank Shell Strengthening		
Shell Area (m^2^) (full-height wrapping)	38.4	38.2
Cost of Carbon Fiber Fabric (300 g/m^2^ UD @ approx. 15 USD/m^2^)	576	573
Cost of Epoxy Resin (approx. 6 USD/m^2^)	230.4	229.2
Cost of Surface Preparation and Application (8 USD/m^2^)	307.2	305.6
Downtime Cost during Application (USD)	Minimal	Minimal
Total Shell Strengthening Cost (USD)	1152	1146

**Figure A1:**
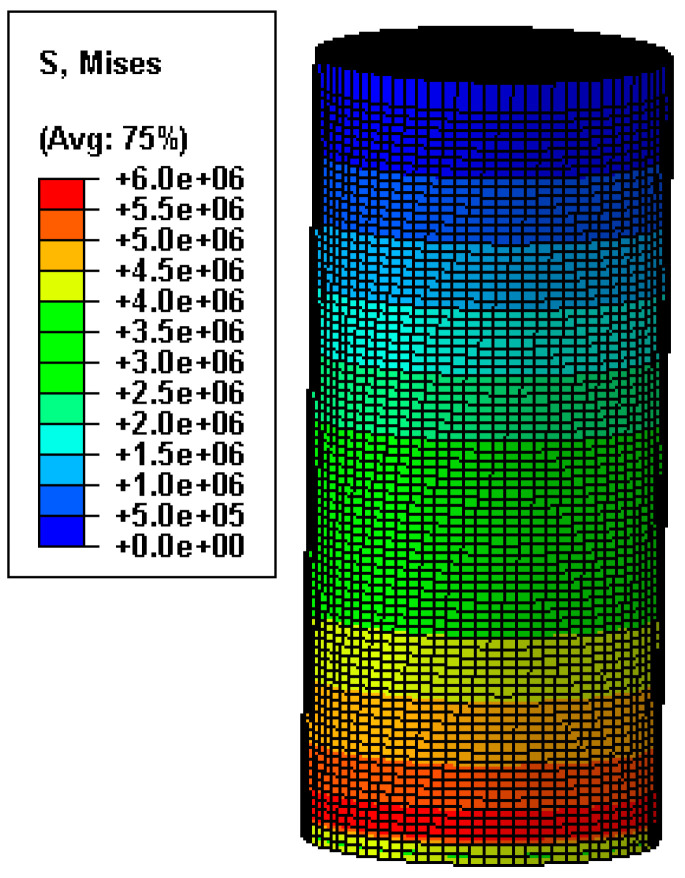
Stress under hydrostatic load without CFRP for tank Type 1.

**Figure A2:**
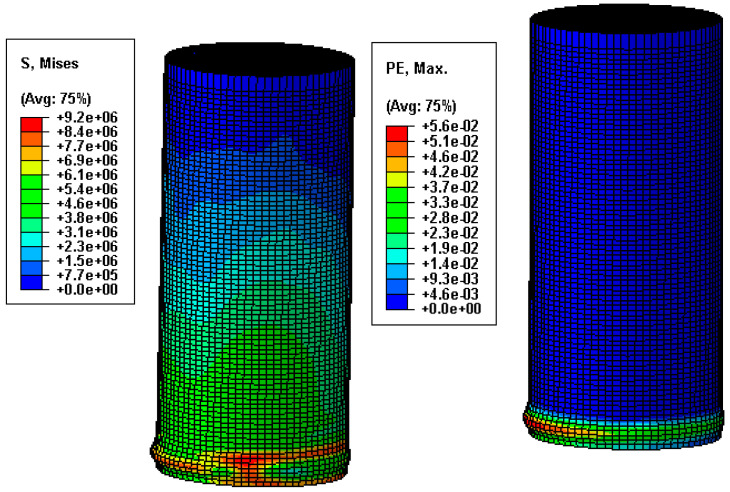
Stress distribution under seismic load without CFRP for tank Type 1.

**Figure A3:**
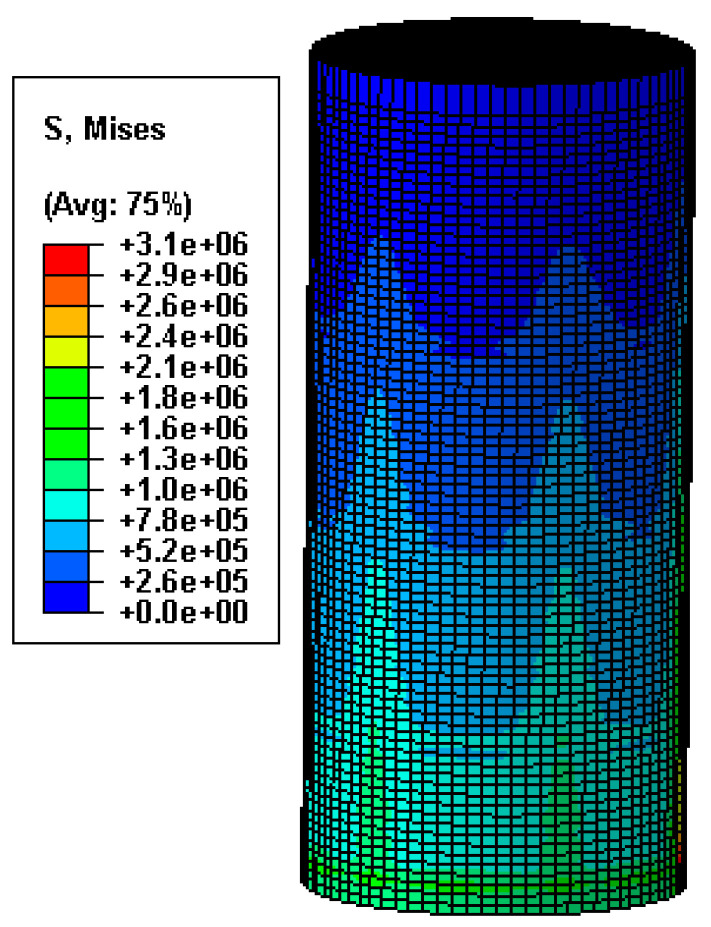
Stress under hydrostatic load with CFRP for tank Type 1.

**Figure A4:**
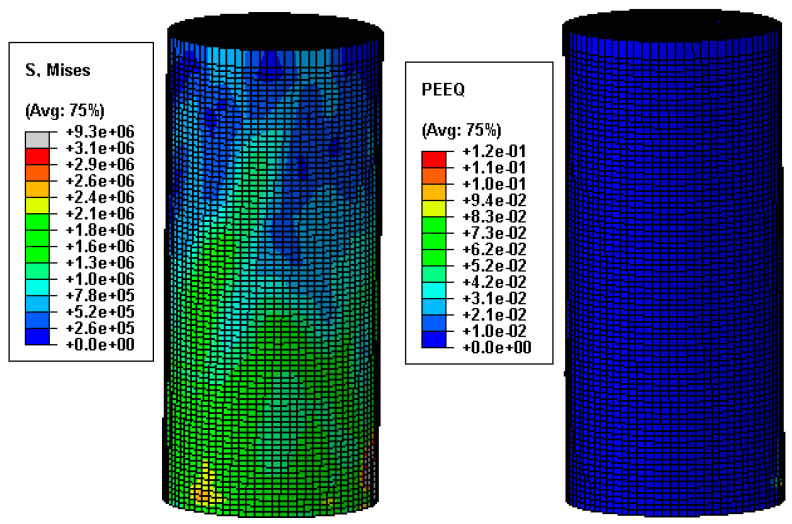
Stress distribution under seismic load with CFRP for tank Type 1.

**Figure A5:**
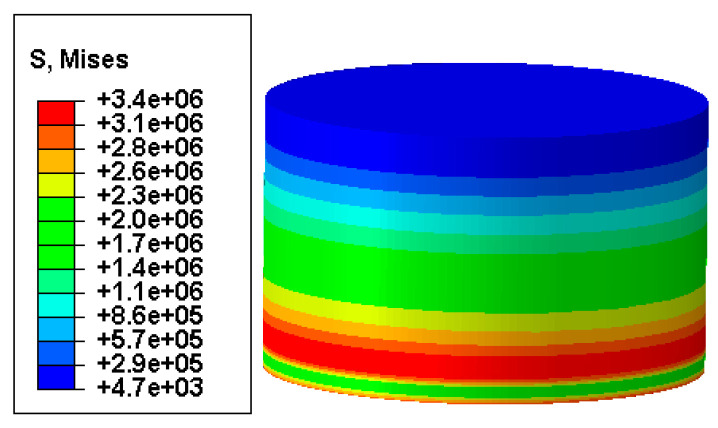
Stress under hydrostatic load without CFRP for tank Type 2.

**Figure A6:**
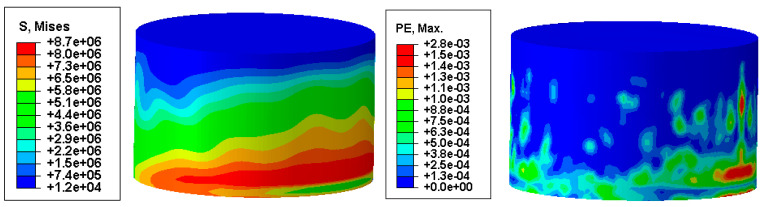
Stress distribution under seismic load without CFRP for tank Type 2.

**Figure A7:**
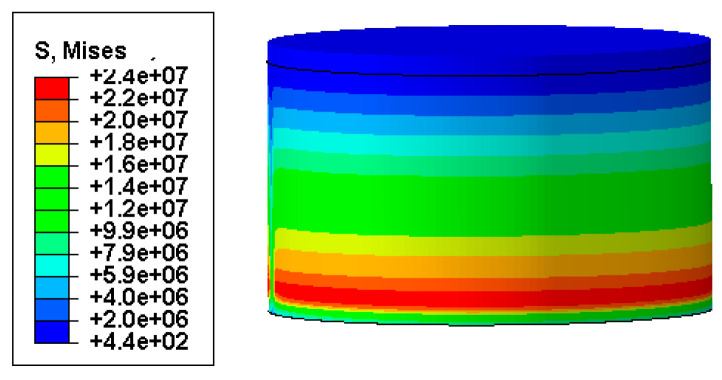
Stress under hydrostatic load with CFRP for tank Type 2.

**Figure A8:**
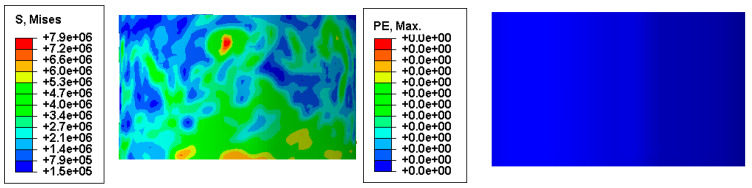
Stress distribution under seismic load with CFRP for tank Type 2.

## Data Availability

All data are available in the paper.

## Abbreviations

The following abbreviations are used in this manuscript:

FRP	Fiber-reinforced polymer
CFRP	Carbon fiber-reinforced polymer
FEA	Finite elements analysis
UV	Ultraviolet
Poly	Polyethylene
MMA	Methyl methacrylate adhesives
Ra	Roughness average (2D)
Sa	Arithmetical mean height (3D)

## Appendix A

This appendix presents supplementary von Mises stress contour plots from the finite element analysis (FEA), complementing the results discussed in Section 5.

Under hydrostatic loading, the tank shell remained structurally sound, with stress levels well below the yield strength of linear low-density polyethylene (LLDPE), as shown in Figure A1 and Figure A5. In contrast, when subjected to seismic loading, the unreinforced models exhibited stress values exceeding the LLDPE yield strength of 16 MPa (Figure A2 and Figure A6), indicating a high probability of localized failure near the base region.

To mitigate these stresses, externally bonded CFRP laminates were applied. The resulting stress contours—shown in Figure A3, Figure A4, Figure A7, and Figure A8—demonstrate a significant reduction in the peak stress values and a more uniform stress distribution across the tank shell. These findings support the effectiveness of CFRP retrofitting in enhancing the structural resilience of polyethylene tanks under dynamic conditions.

These visualizations reinforce the numerical findings by clearly demonstrating the stress mitigation and structural enhancement achieved through CFRP retrofitting under both static and seismic load conditions.

## References

[1] Salakhov I.I., Shaidullin N.M., Chalykh A.E., Matsko M.A., Shapagin A.V., Batyrshin A.Z., Shandryuk G.A., Nifant’ev I.E. (2021). Low-Temperature Mechanical Properties of High-Density and Low-Density Polyethylene and Their Blends. Polymers.

[2] Lai E., Zhao J., Li X., Hu K., Chen G. (2021). Dynamic Responses and Damage of Storage Tanks under the Coupling Effect of Blast Wave and Fragment Impact. J. Loss Prev. Process Ind..

[3] Poly Processing Company PE Tank Life Expectancy. https://tanks.polyprocessing.com/hubfs/images/uploads/PE-Life-Expectancy.pdf.

[4] Vakili M., Showkati H. (2015). Experimental and Numerical Investigation of Elephant Foot Buckling and Retrofitting of Cylindrical Shells by FRP. J. Compos. Constr..

[5] Phan Viet N., Kitano Y., Matsumoto Y. (2020). Experimental Investigations of the Strengthening Effects of CFRP for Thin-Walled Storage Tanks under Dynamic Loads. Appl. Sci..

[6] Owens D.K., Wendt R.C. (1969). Estimation of the Surface Free Energy of Polymers. J. Appl. Polym. Sci..

[7] Forgeway Guide to Bonding Plastics. https://www.forgeway.com/learning/blog/guide-to-bonding-plastics.

[8] Siddika A., Mamun M.A.A., Ferdous W., Alyousef R. (2020). Performances, Challenges and Opportunities in Strengthening Reinforced Concrete Structures by Using FRPs—A State-of-the-Art Review. Eng. Fail. Anal..

[9] Hu L., Li M., Yiliyaer T., Gao W., Wang H. (2022). Strengthening of Cracked DH36 Steel Plates by CFRP Sheets under Fatigue Loading at Low Temperatures. Ocean Eng..

[10] Dassault Systèmes (2010). ABAQUS, Version 6.10.1.

[11] Del Vecchio C., Di Ludovico M., Prota A. (2021). Cost and Effectiveness of Fiber-Reinforced Polymer Solutions for the Large-Scale Mitigation of Seismic Risk in Reinforced Concrete Buildings. Polymers.

[12] Infosys Limited (2020). Carbon Composites: A Cost-Effective Alternative to Metals. White Paper..

[13] Batikha M., Chen J., Rotter J., Teng J. (2009). Strengthening Metallic Cylindrical Shells against Elephant’s Foot Buckling with FRP. Thin-Walled Struct..

[14] Wang D., Song B., Diao S., Wang C., Lu C. (2022). Seismic Effect of Marine Corrosion and CFRP Reinforcement on Wind Turbine Tower. Appl. Sci..

[15] (2020). Welded Tanks for Oil Storage.

[16] Poly Processing Company *Repair of Rotomolded Polyethylene Parts*; Al-Zubi, R., Strong, A.B.; Lampson, M., Eds.; Poly Processing Company: French Camp, CA, USA. https://tanks.polyprocessing.com/hubfs/documents/Repairing-Polyethylene.pdf.

[17] Nicholson D. Repairing Plastic Tanks: Reinforcement Is Key to Long-Term Success. Practical Sailor. 21 November 2018; updated 4 June 2020. https://www.practical-sailor.com/plastics/repairing-plastic-tanks.

[18] Intertronics, IPS Adhesives *Fastening & Bonding Engineering News*, 24 January 2025. https://www.intertronics.co.uk/product/scigrip-sg400lse-mma-methyl-methacrylate-adhesive.

[19] Pereira M.A.R., Galvão I., Costa J.D., Leal R.M., Amaro A.M. (2022). Joining of Polyethylene Using a Non-Conventional Friction Stir Welding Tool. Materials.

[20] Mbuge D.O., Gumbe L.O., Rading G.O. (2013). Analysis of the Weld Strength of the High-Density Polyethylene (HDPE) Dam Liner. Afr. J. Sci. Technol. Sci. Eng. Ser..

[21] Astrouski I., Kudelova T., Kalivoda J., Raudensky M. (2022). Shear Strength of Adhesive Bonding of Plastics Intended for High Temperature Plastic Radiators. Processes.

[22] Chateauneuf A., Raphael W., Pitti R. (2013). Reliability of Prestressed Concrete Structures Considering Creep Models. Struct. Infrastruct. Eng..

[23] Abell D.H. (2011). A Study of the Cause of Failure of Rotationally Molded, High-Density Polyethylene, Sodium Hypochlorite Storage Tanks. Ph.D. Thesis.

[24] Amjadi M., Fatemi A. (2021). Creep Behavior and Modeling of High-Density Polyethylene (HDPE). Polym. Test..

[25] Vijay K., Jayapalan S. (2022). Creep Analysis of Water Tank Made of Polypropylene (PP) and High-Density Polyethylene (HDPE) Polymer Material Using ANSYS Simulation. J. Eng. Res..

[26] Pozhil S.N., Waigaonkar S.D., Chaudhari V.V. (2022). Creep Behaviour of Rotomouldable Grade Materials: A Comparative Study. Polym. Degrad. Stab..

[27] Ibrahim N., Al-Saleh A., Sadiq A. (2023). Texture and Pixel Intensity Characterization-Based Image Segmentation with Morphology and Watershed Techniques. Indones. J. Electr. Eng. Comput. Sci..

[28] Abas M., Awadh M.A., Habib T., Noor S. (2023). Analyzing Surface Roughness Variations in Material Extrusion Additive Manufacturing of Nylon Carbon Fiber Composites. Polymers.

[29] Simunovic G., Svalina I., Simunovic K., Saric T., Havrlisan S., Vukelic D. (2016). Surface Roughness Assessing Based on Digital Image Features. Adv. Prod. Eng. Manag..

[30] (2006). Guidelines for the Surface Preparation of Metals and Plastics Prior to Adhesive Bonding.

[31] Fyfe Co. LLC *TYFO S Epoxy System and CFRP Laminate Properties*; Fyfe Co. LLC: San Diego, CA, USA, 2021. https://www.fyfeco.com.

[32] (2019). Standard Test Method for Apparent Hoop Tensile Strength of Plastic or Reinforced Plastic Pipe by Split-Disk Method.

[33] Saudi Basic Industries Corporation (SABIC) SABIC LLDPE Product Page.

[34] Poly Processing Company *Vertical Tank Capacity Chart—15,500* Gallons. https://tanks.polyprocessing.com/hubfs/tankfiles/PPC_CHART-GALLON-VERT-15500.pdf.

[35] (2015). Standard Specification for Polyethylene Upright Storage Tanks.

[36] Poly Processing Company Repair vs. Replacement: Can You Repair a Polyethylene Tank?. https://blog.polyprocessing.com/blog/repair-vs-replacement-can-you-repair-a-polyethylene-tank.

